# A 2,5-Dihydroxybenzoic Acid–Gelatin Conjugate Inhibits the Basal and Hsp90-Stimulated Migration and Invasion of Tumor Cells

**DOI:** 10.3390/jfb11020039

**Published:** 2020-06-03

**Authors:** Anastasiya V. Snigireva, Oleg S. Morenkov, Yuri Y. Skarga, Alexander V. Lisov, Zoya A. Lisova, Alexey A. Leontievsky, Mariya A. Zhmurina, Viktoria S. Petrenko, Veronika V. Vrublevskaya

**Affiliations:** 1 Institute of Cell Biophysics, Federal Research Center “Pushchino Scientific Center for Biological Research of the Russian Academy of Sciences”, 142290 Pushchino, Moscow Region, Russia; snigireva.s@gmail.com (A.V.S.); morenkov_o@mail.ru (O.S.M.); yyskarga@rambler.ru (Y.Y.S.); mariya100694@gmail.com (M.A.Z.); 79182797935@ya.ru (V.S.P.); 2 Skryabin Institute of Biochemistry and Physiology of Microorganisms, Federal Research Center “Pushchino Scientific Center for Biological Research of the Russian Academy of Sciences”, 142290 Pushchino, Moscow Region, Russia; ssl204@rambler.ru (A.V.L.); lisozoy@gmail.com (Z.A.L.); leont@ibpm.pushchino.ru (A.A.L.)

**Keywords:** 2,5-dihydroxybenzoic acid (2,5-DHBA), 2,5-DHBA–gelatin conjugate, heparin-like polymer, extracellular Hsp90, cell migration and invasion

## Abstract

The extracellular cell surface-associated and soluble heat shock protein 90 (Hsp90) is known to participate in the migration and invasion of tumor cells. Earlier, we demonstrated that plasma membrane-associated heparan sulfate proteoglycans (HSPGs) bind the extracellular Hsp90 and thereby promote the Hsp90-mediated motility of tumor cells. Here, we showed that a conjugate of 2,5-dihydroxybenzoic acid with gelatin (2,5-DHBA–gelatin), a synthetic polymer with heparin-like properties, suppressed the basal (unstimulated) migration and invasion of human glioblastoma A-172 and fibrosarcoma HT1080 cells, which was accompanied by the detachment of a fraction of Hsp90 from cell surface HSPGs. The polymeric conjugate also inhibited the migration/invasion of cells stimulated by exogenous soluble native Hsp90, which correlated with the inhibition of the attachment of soluble Hsp90 to cell surface HSPGs. The action of the 2,5-DHBA–gelatin conjugate on the motility of A-172 and HT1080 cells was similar to that of heparin. The results demonstrate a potential of the 2,5-DHBA–gelatin polymer for the development of antimetastatic drugs targeting cell motility and a possible role of extracellular Hsp90 in the suppression of the migration and invasion of tumor cells mediated by the 2,5-DHBA–gelatin conjugate and heparin.

## 1. Introduction

The majority of deaths from cancer are associated with the metastatic dissemination of tumor cells to various organs rather than to the expansion of the primary tumor. The migration and invasion of tumor cells are the key processes of metastasis necessary for the intrusion of malignant tumor cells into adjacent tissues, intravasation into the lymphatic or vascular circulation, and subsequent extravasation [1]. A promising approach to gain control over the metastasis of tumor cells is targeting the motility of tumor cells. Heparan sulfate proteoglycans (HSPGs) are glycoproteins located at the plasma membrane and in the extracellular matrix. HSPGs consist of the core protein with several covalently attached heparan sulfate (HS) glycosaminoglycan chains [2,3]. Owing to the structural diversity of HS moieties, cell surface-associated HSPGs are capable of interacting with a variety of biologically active ligands (growth factors, cytokines, etc.) and modulating numerous functions of malignant cells involved in the growth and metastasis of the tumor [2,3]. Therefore, the inhibition of the binding of cell surface HSPGs with their ligands is considered as a promising approach to the development of novel drugs with anticancer activity [4]. Heparin, a highly sulfated glycosaminoglycan related to the HS moieties of HSPGs [2,5], and its analogues mimic the HS moieties and compete with cell surface-associated HSPGs for binding to biologically active ligands that exhibit heparin-binding properties, which results in their anticancer activity [5,6,7]. The anticoagulant property of heparin restrains its application as an anticancer agent; therefore, non-anticoagulant heparin analogues with anticancer activity have been developed [8,9,10]. The effects of heparin and its analogues on cancer cells are very complex. Heparins, heparin mimetics, and heparin-like substances suppress tumor growth, retard metastasis by inhibiting the invasion of tumor cells, and affect angiogenesis [5,6,7,9,11]. Heparins and heparin-like substances have been shown to inhibit heparinase, the tissue factor, P- and L-selectins; they affect the interactions of malignant tumor cells with integrins and modulate the composition of the extracellular matrix [5,6,7,12,13,14,15].

Heat shock protein 90 (Hsp90) is a molecular chaperone that performs vital housekeeping functions in cells, participating in the control of the folding, stability, activation, functioning, and turnover of a diversity of client proteins [16]. Hsp90 is also expressed on the plasma membrane of a number of normal and cancer cells and is actively secreted by tumor cells [17,18,19,20,21,22,23]. In tumor cells, the expression of plasma membrane-associated Hsp90 and the secretion of Hsp90 are elevated and correlate with the metastatic properties of these cells [17]. Extracellular Hsp90 enhances cell motility and thereby stimulates migration, invasion, and metastasis of cancer cells [17,18,19,20,21,22,23,24]. It has been demonstrated that cell-impermeable substances inhibiting membrane-associated extracellular Hsp90 (Hsp90-specific antibodies, DMAG-N-oxide, STA-12-7191, and others) reduce cell migration, invasion, and tumor dissemination [19,21,22,24,25], whereas soluble Hsp90 enhances cell migration and invasion [26,27,28]. Thus, both forms of extracellular Hsp90, plasma membrane-associated and soluble Hsp90, participate in the promotion of cell motility. Therefore, the extracellular Hsp90 is considered as a novel chemotherapeutic target for antimetastatic drugs. Earlier, we found that HSPGs associated with the cell surface participate in the binding of soluble extracellular Hsp90 and the anchoring of Hsp90 to the cell plasma membrane [29]. It was also demonstrated that cell surface-associated HSPGs facilitate the interaction of Hsp90 with surface receptors, which is required for the efficient signaling related to cell motility and further migration and invasion of cells [30]. Hence, cell-impermeable natural and artificial compounds interfering with the binding of Hsp90 to cell surface HSPGs can be considered as novel drugs targeting the invasion and migration of cancer cells; expectedly, these substances would not affect the vital functions of intracellular Hsp90. Recently we synthesized a conjugate of 2,5-dihydroxybenzoic acid (2,5-DHBA) with gelatin using the enzyme laccase and showed that, similar to heparin, the conjugate impairs the binding of alphaherpesviruses to HSPGs present on the plasma membrane of susceptible cells [31]. We assumed that the 2,5-DHBA–gelatin conjugate, which possesses heparin-like properties, could interfere with the adsorption of extracellular Hsp90 to cells, thereby affecting the Hsp90-mediated motility of cancer cells; in this case, the conjugate, due to its cell-impermeability, will not impair the function of intracellular Hsp90. In this report, we demonstrated that the 2,5-DHBA–gelatin polymeric conjugate was not cytotoxic to two tumor cell lines, human glioblastoma A-172 and fibrosarcoma HT1080, and did not affect their proliferation. The conjugate, similar to heparin, inhibited the basal migration and invasion of cells, which correlates with the detachment of cell surface-bound Hsp90 from cell surface HSPGs. The polymeric conjugate also inhibited the binding of extracellular soluble Hsp90 to cell surface HSPGs, as well as the migration and invasion of cells stimulated by soluble extracellular Hsp90.

## 2. Results

### 2.1. The 2,5-DHBA–Gelatin Conjugate Exhibited No Cytotoxic and Antiproliferative Activities Against Tumor Cell Lines

**Figure 1:**
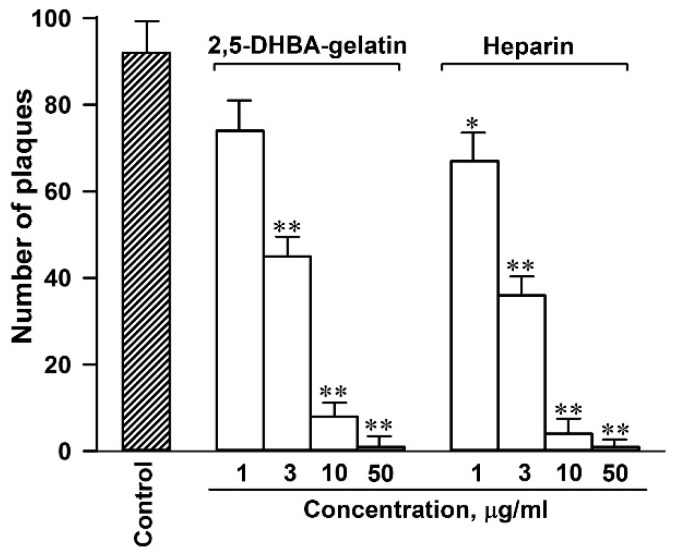
The 2,5-dihydroxybenzoic acid (2,5-DHBA)–gelatin conjugate inhibited the adsorption of pseudorabies virus (PRV) to cells. The degree of inhibition was determined by the plaque assay. The mean values of four to six repeats ± SD are presented. The statistical difference from control cells: * *p* < 0.05; ** *p* < 0.01.

The synthesis of the conjugate was performed by oxidizing 2,5-DHBA in the presence of gelatin catalyzed by laccase [31]. The conjugate, a dark brown polymer owing to the presence of the 2,5-DHBA chromophore, was soluble in water and was sterilized by autoclaving for cell culture experiments. In accordance with our previous results [31], the conjugate, similar to heparin, inhibited the adsorption of the pseudorabies virus (PRV), a member of the *Alphaherpesvirinae* subfamily, to cell surface HSPGs (IC_50_ 5.0–2.5 µg/mL), thereby exhibiting the heparin-like activity (Figure 1).

The 2,5-DHBA–gelatin conjugate did not exhibit direct toxicity to cells of both cancer cell lines and did not affect the proliferation of cells at concentrations in the range of 10–1000 µg/mL (Table 1). Heparin, as well as dermatan sulfate A and chondroitin sulfate, two other sulfated glycosaminoglycans, were also not toxic to cells and did not impair the proliferation of cells. Geldanamycin, a well-known cell-permeable inhibitor of intracellular Hsp90, which causes a simultaneous degradation of many Hsp90 client proteins followed by the suppression of the growth and killing of cancer cells [32], demonstrated strong cytotoxic and antiproliferative activities, which indicated the validity of the MTT assay in the analysis of the cytotoxic and antiproliferative properties of the polymeric conjugate.

### 2.2. The 2,5-DHBA–Gelatin Conjugate Decreased the Basal Migration and Invasion of Cells In Vitro

**Figure 2:**
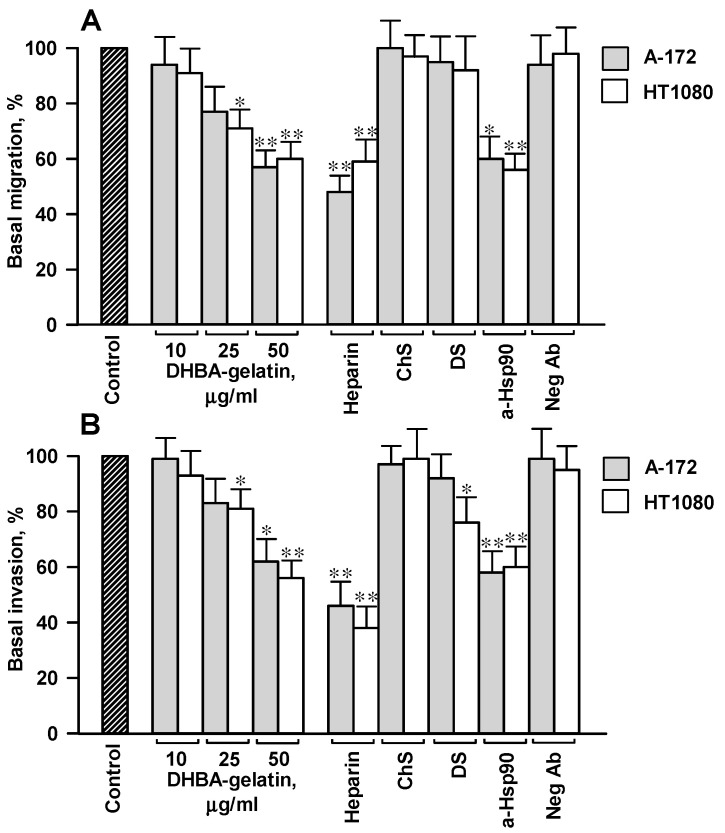
The 2,5-DHBA–gelatin conjugate inhibited the basal unstimulated cell migration and invasion. (**A**,**B**) The migration/invasion of cells was determined in the presence of 2,5-DHBA–gelatin at concentrations of 10–50 μg/mL and of heparin, dermatan sulfate (DS), chondroitin sulfate A (ChS), and polyclonal anti-heat shock protein 90 (Hsp90) antibodies (concentration of 50 μg/mL for all reagents). The migration/invasion of untreated cells (control) was assumed to be 100%. The mean values of three to five repeats ± SD are presented. Asterisks indicate the statistical difference from control cells: * *p* < 0.05, ** *p* < 0.01.

To evaluate the basal migration and invasion of cells without external stimuli, A-172 and HT1080 cells were serum-starved and seeded in transwell inserts in DMEM-BSA (without external stimuli) in the presence of the 2,5-DHBA–gelatin conjugate at concentrations of 10–50 μg/mL. The conjugate dose-dependently inhibited the basal cell migration and invasion. It reduced the unstimulated migration and invasion of cells by 30%–45% at a concentration of 50 μg/mL (Figure 2). The basal unstimulated migration and invasion of cells decreased by 40%–60% in the presence of heparin (50 μg/mL). Dermatan sulfate A and chondroitin sulfate, two other sulfated glycosaminoglycans, at a concentration of 50 μg/mL did not impair or insignificantly affect the basal migration and invasion of cells (Figure 2). Altogether, the results demonstrated that the 2,5-DHBA–gelatin conjugate, similarly to heparin, profoundly suppressed the basal migration and invasion of A-172 and HT1080 cells, while these processes were not affected or were only slightly impaired by dermatan sulfate and chondroitin sulfate A.

### 2.3. The 2,5-DHBA–Gelatin Conjugate Detached Hsp90α And Hsp90β from the Cell Surface

**Figure 3:**
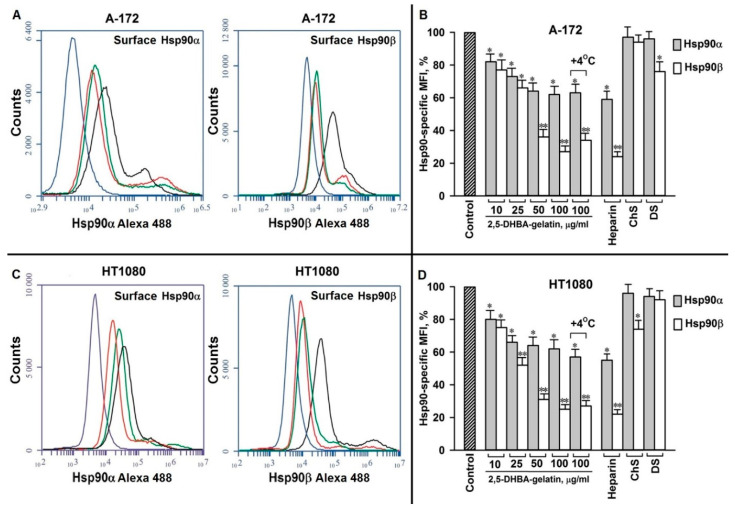
The 2,5-DHBA–gelatin conjugate dissociated Hsp90α and Hsp90β from the cell plasma membrane. Cells were treated with 2,5-DHBA–gelatin at concentrations of 10–100 μg/mL and with heparin, dermatan sulfate (DS), or chondroitin sulfate A (ChS) (a concentration of 50 μg/mL for all substances). Incubation in all experiments was performed for 1 h at 37 °C, except one experiment in which cells were incubated at 4 °C (indicated in the graph). After the treatment, the expression of Hsp90 isoforms on the plasma membrane was determined by flow cytometry using Hsp90α- and Hsp90β-specific antibodies. (**A**,**C**) Representative flow cytometry histograms for A-172 and HT1080 cells. Control (untreated) cells (black lines), 2,5-DHBA–gelatin-treated cells (red lines), and cells treated with heparin (blue lines) were probed with antibodies directed to Hsp90α and Hsp90β; control cells were also probed with the isotype control antibody (green lines). (**B**,**D**) Quantification of membrane-associated Hsp90α and Hsp90β levels after different treatments. The Hsp90 isoform-specific mean fluorescence intensity (MFI) are presented; the MFIs of control cells were assumed to be 100%. The mean values of three to five repeats ± SD are presented. The representative results from two to four experiments are presented.

To address a possible role of cell surface Hsp90s in the 2,5-DHBA–gelatin-mediated decrease in basal migration and invasion, we analyzed the effect of 2,5-DHBA–gelatin polymer on the level of surface-associated Hsp90α and Hsp90β, since cell surface Hsp90s are involved in sustaining the unstimulated basal migration and invasion of cells [19,21,22,24,25]. Here, we also observed that rabbit polyclonal antibodies specific for Hsp90α and Hsp90β decreased the basal cell migration/invasion by 30%–40% compared to control negative antibodies, confirming the role of Hsp90s associated to the cell surface in cell motility (Figure 2). As we have shown earlier, a part of Hsp90s at the cell surface is bound to HSPGs, which represent a heparin-sensitive fraction of Hsp90 [29]. HSPG-associated Hsp90s participate in efficient motility-related signaling, and they further cell migration and invasion [30]. Since the 2,5-DHBA–gelatin conjugate exhibited heparin-like properties, we anticipated that it would disrupt the interaction of Hsp90s with cell surface HSPGs, which may lead to a decrease in cell motility. Indeed, the polymeric conjugate dissociated the fraction of both isoforms of Hsp90 from the surface of cells of both cell cultures (Figure 3).

The conjugate exerts its effect on the cell surface Hsp90 isoforms in a concentration-dependent manner; the levels of surface-associated Hsp90α and Hsp90β were reduced by the polymer even at a concentration of 10 μg/mL. At concentrations of 50–100 μg/mL, the conjugate decreased the level of Hsp90α and Hsp90β associated to cell surface by 25%–35% and 40%–70%, respectively. The effect of 2,5-DHBA–gelatin on the cell surface Hsp90 was comparable to that of heparin—at a concentration of 50 μg/mL, it decreased the levels of Hsp90α and Hsp90β by 30%–55% and 50%–75%, respectively (Figure 3). As opposed to the 2,5-DHBA–gelatin polymer and heparin, dermatan sulfate and chondroitin sulfate A did not detach Hsp90α and Hsp90β from the cell surface (Figure 3). The effect of the conjugate on the level of Hsp90α and Hsp90β on the surface at 37 °C was nearly the same as at 4 °C, a temperature at which protein translocation across the membrane, secretion, and internalization from the plasma membrane are inhibited. The results demonstrated that the synthesized 2,5-DHBA–gelatin polymeric conjugate, similar to heparin, detached the Hsp90 fraction associated with cell surface HSPGs, which correlated with the 2,5-DHBA–gelatin-mediated decrease in the basal migration and invasion of cells.

### 2.4. The Migration and Invasion of Cells Stimulated by Exogenous Hsp90 Was Reduced by the 2,5-DHBA–Gelatin Conjugate

**Figure 4:**
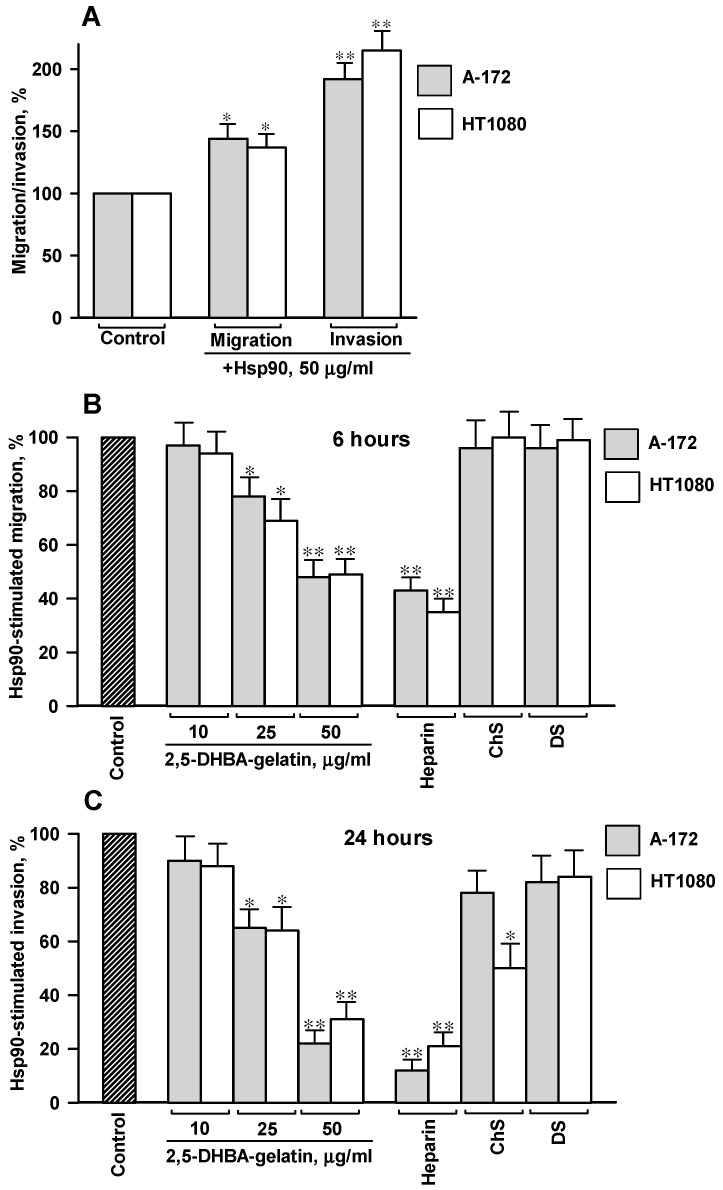
Treatment of cells with 2,5-DHBA–gelatin decreased the Hsp90-mediated stimulation of migration and invasion of cells. (**A**) Soluble native bovine Hsp90 stimulated cell motility. Cell migration/invasion was expressed in percent; the migration/invasion of control cells without Hsp90 was taken as 100%. (**B**) Hsp90-induced stimulation of cell migration. (**C**) Hsp90-induced stimulation of cell invasion. The degree of stimulation of cell migration/invasion by soluble Hsp90 was determined in the presence of the conjugate in the medium at concentrations of 10–50 μg/mL or in the presence of heparin, dermatan sulfate (DS), and chondroitin sulfate A (ChS) in the medium (50 μg/mL for all substances). The stimulation of migration/invasion induced by soluble Hsp90 was expressed in percent relative to control cells. (**A**–**C**) The mean values of three to five repeats ± SD are presented. Statistical difference from control cells: * *p* < 0.05, ** *p* < 0.01.

Extracellular soluble Hsp90 is a well-recognized promotility factor that stimulates migration, invasion, and metastasis of tumor cells [17,18,19,20,21,22,23,24]. Native purified bovine Hsp90 (50 μg/mL) was used for the stimulation of migration and invasion of cells. Cell migration and invasion were evaluated after 6 and 24 h, respectively. Native Hsp90 enhanced the migration of HT1080 and A-172 cells by 30%–60% and the invasion of cells by 80%–160% (Figure 4A).

The addition of the conjugate to the medium dose-dependently suppressed the migration/invasion of cells stimulated by soluble Hsp90 (Figure 4). The polymeric conjugate at a concentration of 50 μg/mL reduced the Hsp90-mediated stimulation of migration by 40%–55% and the Hsp90-mediated stimulation of invasion by 65%–80%, as compared to the stimulation of cell motility without 2,5-DHBA–gelatin. The effect of heparin on the Hsp90-induced stimulation of cell migration was somewhat higher than that of the 2,5-DHBA–gelatin conjugate; heparin (50 μg/mL) decreased the stimulation of migration of cells by 50%–70% and invasion of cells by 80%–90%. By contrast, two other sulfated glycosaminoglycans, dermatan sulfate and chondroitin sulfate A, did not affect the Hsp90-induced stimulation of cell migration (Figure 4B). Dermatan sulfate and chondroitin sulfate A diminished the Hsp90-mediated stimulation of invasion of HT1080 cells by 40%–50% and 15%–20%, respectively; both sulfated proteoglycans reduced the Hsp90-induced stimulation of invasion by 10%–30% in A-172 cells (Figure 4C). Altogether, the results clearly indicated that the stimulatory effect of soluble Hsp90 on the migration and invasion of A-172 and HT1080 cells was strongly impaired by the 2,5-DHBA–gelatin conjugate. The effects of the 2,5-DHBA–gelatin conjugate were similar to those of heparin, while dermatan sulfate and chondroitin sulfate A did not alter the Hsp90-stimulated migration and invasion of A-172 and HT1080 cells or reduced these processes to a much lesser extent as compared to heparin and 2,5-DHBA–gelatin.

### 2.5. The 2,5-DHBA–Gelatin Conjugate Specifically Inhibited the Binding of Soluble Hsp90 to the Cell Surface

**Figure 5:**
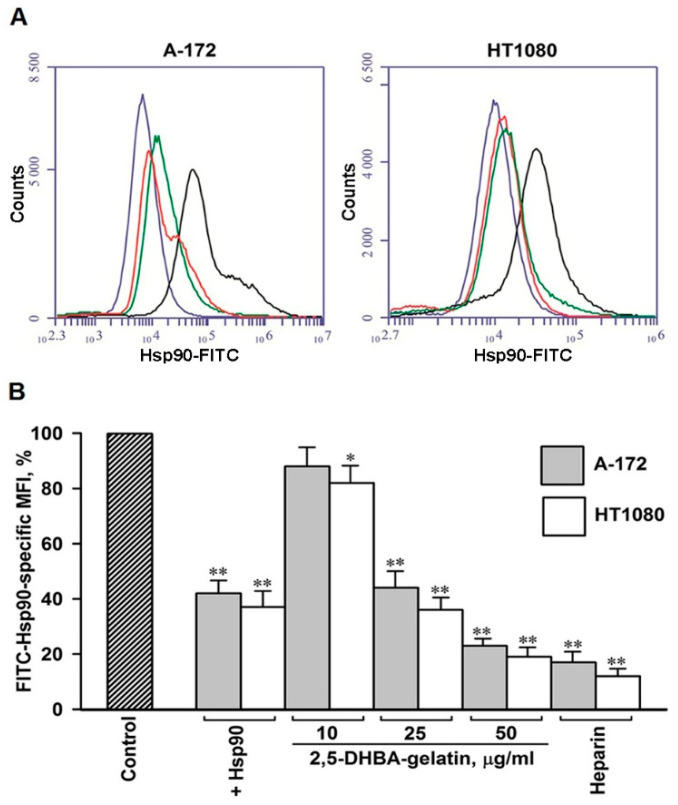
The 2,5-DHBA–gelatin polymeric conjugate impaired the binding of FITC-labelled native Hsp90 to cells. Cells were incubated with Hsp90–FITC for 1 h at 4 °C in the presence of the 2,5-DHBA–gelatin conjugate at concentrations in the range of 10–50 μg/mL and heparin (50 μg/mL). The binding of Hsp90–FITC was analyzed by flow cytometry. (**A**) Representative flow cytometry histograms. Cells incubated with Hsp90–FITC were treated with 2,5-DHBA–gelatin (green lines) and heparin (red lines); untreated (control) cells are represented as black lines, whereas blue lines represent cells incubated without Hsp90–FITC (autofluorescence). (**B**) Quantification of the binding of Hsp90–FITC to cells. The MFI of 2,5-DHBA–gelatin-treated and untreated control cells is presented; the MFIs of control untreated cells were taken as 100%. The mean values of three to five repeats ± SD are presented. Asterisks indicate the statistical difference from control cells: * *p* < 0.05, ** *p* < 0.01.

Soluble Hsp90s of the extracellular milieu bind to cell surface receptors and induce signaling related to motility in normal and cancer cells, which enhances their migration and invasion [20,24,33,34,35,36,37,38,39,40,41]. Earlier we have demonstrated that cell surface HSPGs participate in the binding of Hsp90α and Hsp90β to the cell plasma membrane and that the HSPG-mediated binding of Hsp90s to cells plays a significant role in the Hsp90-stimulated motility [29,30]. Here, we determined how the 2,5-DHBA–gelatin polymeric conjugate affects the attachment of soluble Hsp90 to the surface of cells. We showed that FITC-labeled soluble Hsp90 efficiently bound at 4 °C to the surface of both cell types (Figure 5). The binding of FITC-labeled soluble Hsp90 to cells was inhibited by an excess of unlabeled Hsp90, suggesting the specificity of the attachment of Hsp90 to cells. The binding of FITC-labeled Hsp90 to the cell surface was dose-dependently inhibited by the 2,5-DHBA–gelatin conjugate (Figure 5). The extent of the inhibition of Hsp90 binding to cells at the concentrations of the conjugate of 50 and 100 μg/mL was approximately the same at 2,5-DHBA–gelatin concentrations of 50 and 100 μg/mL. The effect of the polymeric conjugate on the direct binding of Hsp90 was similar to that of heparin (Figure 5). The data obtained clearly indicated that the 2,5-DHBA–gelatin conjugate affected the direct binding of extracellular soluble Hsp90 to cell surface HSPGs.

## 3. Discussion

A promising approach to the production of synthetic polymers possessing biological activity, including anticancer activity, is the grafting of different compounds onto a polymeric carrier. Some synthetic polymers with different structures were shown to possess antitumor activity [42,43,44,45,46]. Polymers exhibiting anticancer activity are often polyanions with sulfonate, phosphate, and carboxylic groups [47]. The multivalent interactions of polymers with receptors located on the cell surface are considered to play a critical role in their activity. The grafting of compounds with sulfonate, phosphate, and carboxylate groups onto a polymeric carrier leads to the creation of synthetic polymers that imitate the functions of natural bioactive polymers. Earlier, we showed that a conjugate of 2,5-dihydroxybenzoic acid with gelatin had heparin-like properties and produced a strong antiviral effect on alphaherpesviruses; similar to heparin, it suppressed the viral infection by inhibiting the adsorption of the viruses to HSPGs located on the surface of target cells [31]. Because a variety of heparin-like substances exhibit anticancer activity [4,5,6,7], we herein examined the effects of the 2,5-DHBA–gelatin polymeric conjugate on the proliferation, migration, and invasion in vitro of two cancer cell lines—human glioblastoma A-172 and fibrosarcoma HT1080 cells.

We demonstrated that the 2,5-DHBA–gelatin conjugate at concentrations up to 1000 µg/mL was nontoxic to both cancer cell lines and neither inhibited nor stimulated cell proliferation. The cell-permeable inhibitor of intracellular Hsp90 geldanamycin [32] strongly decreased the viability and proliferation of cells of both cell lines at nanogram concentrations, indicating that the MTT assay is suitable for evaluating the cytotoxic and antiproliferative activity of the conjugate. Earlier, we also observed that the 2,5-DHBA–gelatin conjugate did not exhibit cytotoxicity and antiproliferative activity for Vero, MDBK, and BHK-21 cell lines [31]. It is worth noting that the effects of heparin and glycosaminoglycans on cell proliferation vary considerably. The action of glycosaminoglycans on cell growth is cell-type specific, often differs at different concentrations of the substances, and depends on the type of glycosaminoglycan [4,5,6,7,48,49,50,51,52]. The lack of toxicity of the 2,5-DHBA–gelatin conjugate is presumably attributable to its polymeric hydrophilic nature, which precludes the efficient translocation of the polymer across the cell plasma membrane. On the contrary, geldanamycin and other inhibitors of intracellular Hsp90 are cell-permeable and severely impair the function of Hsp90 inside the cell, which leads to the disturbance of many cell functions and strong cytotoxicity [32].

Heparins and heparin-like substances are known to suppress cell migration and invasion [4,5,6,7,11]. We observed that the 2,5-DHBA–gelatin conjugate also inhibited the basal migration/invasion of tumor cells in vitro in the absence of external stimuli. Its effects on basal unstimulated cell migration/invasion were qualitatively and quantitatively similar to those of heparin. On the contrary, the basal migration/invasion of cells was not impaired by dermatan sulfate and chondroitin sulfate A, suggesting the involvement of HSPGs on the cell surface in the 2,5-DHBA–gelatin-mediated inhibition of basal migration/invasion. Since we performed the experiments in DMEM-BSA, a medium that essentially lacks growth and promotility factors, the 2,5-DHBA–gelatin-mediated decrease in basal cell motility cannot be explained by the interference of the conjugate with the binding of exogenous serum-derived growth/promotility factors to cells. We suggested that the 2,5-DHBA–gelatin polymeric conjugate and heparin may affect HSPG-bound motility-related autocrine and paracrine factors produced by tumor cells [53]. Indeed, in parallel with the impairment of basal cell migration and invasion, 2,5-DHBA–gelatin dissociated from the cell surface a fraction of cell surface HSPG-associated Hsp90 (both Hsp90α and Hsp90β isoforms), a well-recognized promotility factor [17,18,19,20,21,22,23,24]. Using Hsp90-specific antibodies, we demonstrated that cell surface-associated Hsp90s play a role in sustaining the basal cell motility (Figure 2), which corresponds to the earlier published results [19,20,21,22,24,25]. The effects of 2,5-DHBA–gelatin on the levels of cell surface Hsp90α and Hsp90β and on the basal migration/invasion of cells were similar to those of heparin, but not of dermatan sulfate and chondroitin sulfate A. Cell surface HSPGs are known to participate in the attachment of Hsp90 to the cell plasma membrane and facilitate the interaction of Hsp90s with surface receptors, which is necessary for the efficient motility-related signaling, further cell migration, and invasion [29,30]. Thus, the detachment of cell surface HSPG-associated Hsp90s by the 2,5-DHBA–gelatin polymeric conjugate or by heparin may lead to signaling that does not suffice for the activation of cell motility. Although it is tempting to explain the effects of the conjugate on cell motility exclusively by its action on HSPG-associated Hsp90s, we cannot exclude the fact that the 2,5-DHBA–gelatin conjugate may affect the action of other growth/motility factors associated with cell surface HSPGs.

Exogenous soluble native Hsp90 stimulated the migration and invasion of both tumor cell lines, which is consistent with the results reported in [17,18,19,20,21,22,23,24]. The 2,5-DHBA–gelatin conjugate not only impaired the basal unstimulated cell migration and invasion but also suppressed the migration/invasion of cells promoted by soluble Hsp90. The inhibitory effects were similar to those of heparin, while dermatan sulfate and chondroitin sulfate A, as in the case of basal migration/invasion, reduced the Hsp90-induced cell migration/invasion to a much lesser extent. To address a possible mechanism of action of the 2,5-DHBA–gelatin conjugate on the Hsp90-mediated cell motility, we evaluated the influence of the polymer on the binding of the Hsp90–FITC conjugate to live HT1080 and A-172 cells using the method of flow cytometry detection. We demonstrated that the 2,5-DHBA–gelatin polymeric conjugate, similarly to heparin, hampers the direct binding of extracellular soluble Hsp90 to cell surface HSPGs, which presumably impairs the cell motility-related Hsp90 signaling. A limitation of the method used was that it determined the integral 2,5-DHBA–gelatin-sensitive binding of soluble Hsp90 to cells. A more detailed investigation is required to estimate the binding of the extracellular Hsp90 to different representatives of surface HSPGs.

**Figure 6:**
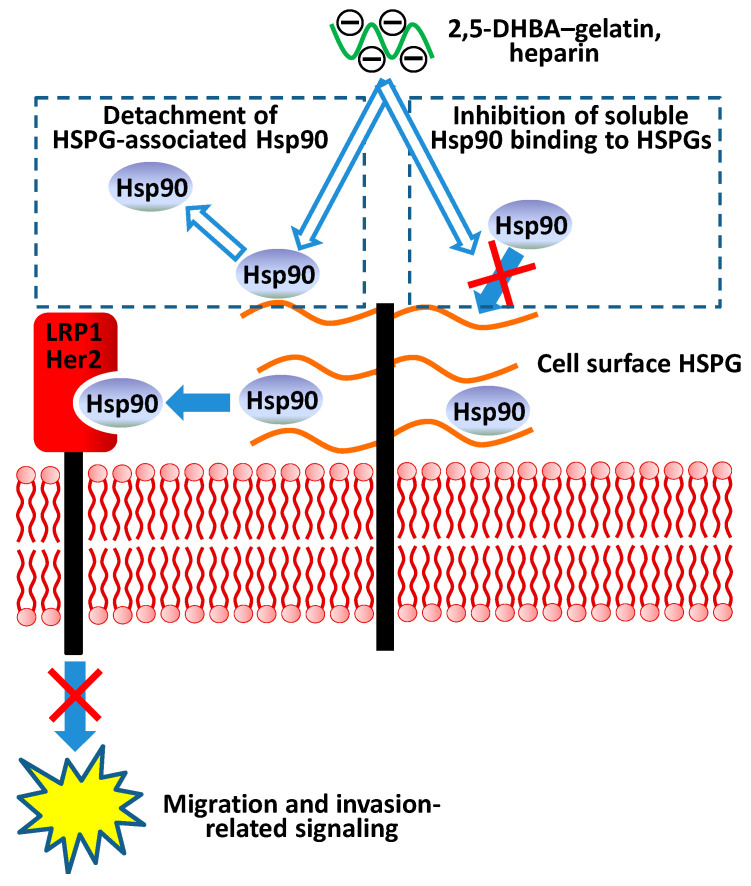
A hypothetical model of the action of the 2,5-DHBA–gelatin conjugate, heparin, and heparin-like substances on the Hsp90-mediated migration and invasion of tumor cells. Heparan sulfate (HS) glycosaminoglycan chains of cell surface heparan sulfate proteoglycans (HSPGs) bind with extracellular Hsp90s and facilitate Hsp90-mediated signaling via cell receptors LRP1 and Her2 associated with cell migration and invasion. The 2,5-DHBA–gelatin conjugate and heparin compete with HS for the binding with extracellular Hsp90, which results in the detachment of HSPG-associated Hsp90 from the cell surface HSPGs and the inhibition of binding of soluble Hsp90 to cell surface HSPGs. Both processes lead to the impairment of Hsp90-mediated motility-related signaling, which results in an inhibition of the migration and invasion of tumor cells.

Thus, on the basis of our data, a possible mechanism of action of the 2,5-DHBA–gelatin conjugate, and probably of heparins and heparin-like polymers on basal and Hsp90-stimulated migration/invasion of tumor cells can be proposed (Figure 6). The mechanism includes the detachment of HSPG-associated Hsp90 from the cell surface (basal migration/invasion) and the interference of the conjugate with the binding of extracellular soluble Hsp90 to surface HSPGs. Because cell surface HSPGs take part in efficient Hsp90-mediated signaling associated with cell motility [30], the impairment of the binding/association of extracellular Hsp90 to cell surface HSPGs results, as a consequence, in reduced migration and invasion of cells. Therefore, since the 2,5-DHBA–gelatin polymer impairs the Hsp90-mediated cell motility, it can be considered as a promising substance for the development of anticancer drugs with antimetastatic activity. It is worth noting that the synthetic polymer even at high concentrations does not exhibit cytotoxicity and antiproliferative activity against cancer cells, as well as against normal cells [31], which is a prerequisite for the development of drugs on its basis. The anticoagulant activity of heparin-like substances is known to limit their application as anticancer drugs [8,9,10]. Therefore, further studies are needed for the determination of the anticoagulant activity of 2,5-DHBA–gelatin and the evaluation of its effects on the growth and metastasis of cancer cells in vivo.

Even at high concentrations, the 2,5-DHBA–gelatin conjugate and heparin were not able to remove cell surface-attached Hsp90α and Hsp90β completely, so that a substantial fraction of cell surface-associated Hsp90 (60%–70% of Hsp90α and 20%–30% of Hsp90β) was insensitive to the substances. The 2,5-DHBA–gelatin- and heparin-resistant fraction of Hsp90 is assumed to associate with non-HSPGs surface Hsp90 receptors (for example, LRP1) [33,34,41]. The conjugate and heparin did not completely abolish the stimulatory effect of exogenous Hsp90 on cell migration and invasion, which suggests that soluble Hsp90 is capable of stimulating of migration and invasion of cells without its association with HSPGs. Thus, extracellular Hsp90 possesses a dual promotility activity—HSPG-dependent activity that can be inhibited by heparin and heparin-like substances and HSPG-independent activity related to the interaction of extracellular Hsp90 with cell surface receptors in an HSPG-independent manner.

It has been shown earlier through using specific antibodies that both isoforms of cell surface Hsp90, Hsp90α and Hsp90β, stimulate cell migration [22,54]. As far as soluble extracellular Hsp90 is concerned, Hsp90α is considered to be a principal stimulator of migration and invasion of cells, whereas the impact of Hsp90β in these processes seems to be less important [26,33,34,37,41]. In this study, we did not examine the influence of 2,5-DHBA–gelatin on the Hsp90α and Hsp90β isoforms separately. Therefore, a particular role of each isoform of membrane-associated and soluble Hsp90 in the 2,5-DHBA–gelatin-mediated suppression of basal and Hsp90-stimulated migration/invasion remains unclear and requires further investigation.

It is noteworthy that the structures of heparin and the synthetic 2,5-DHBA–gelatin conjugate differ greatly. We have shown earlier that the 2,5-DHBA–gelatin conjugate contains free carboxyl groups, which makes it a polyanion [31]. We suppose that the electrostatic surface of the polymers is similar to that of the HS moiety of cell surface HSPGs; heparin and 2,5-DHBA–gelatin compete with HSPGs associated with the cell surface for binding to extracellular Hsp90 and its anchoring to the plasma membrane, which leads to the impairment of Hsp90-mediated motility-related signaling. We expect that the synthetic polymer, which mimics heparin, would also affect the action of growth factors and morphogens whose activity is positively modulated by cell surface HSPGs [2,55].

To summarize, our data indicate that the synthetic 2,5-DHBA–gelatin polymer exhibiting heparin-like properties inhibits the basal migration/invasion of cells without the external stimuli and the cell migration/invasion stimulated by the promotility factor Hsp90. The inhibition of basal cell migration/invasion correlates with the detachment of Hsp90 from cell surface HSPGs caused by the polymer; the inhibition of migration/invasion stimulated by soluble Hsp90 correlates with the reduction in the binding of soluble extracellular Hsp90 to the plasma cell membrane in the presence of the conjugate. The effects of the 2,5-DHBA–gelatin polymeric conjugate on basal and Hsp-90-stimulated cell migration/invasion are similar to those of heparin. The 2,5-DHBA–gelatin conjugate is easy to synthesize by laccase-mediated oxidative coupling and may be considered as a promising synthetic polymer with anticancer activity.

## 4. Materials and Methods

The study was conducted in accordance with the Declaration of Helsinki; the protocol received approval by the Bioethics Committee of the Institute of Cell Biophysics of the Russian Academy of Sciences (Approval ID: 2018-11, date: 2018-4-5).

### 4.1. Materials

Fetal bovine serum (FBS), Dulbecco’s modified Eagle’s medium (DMEM), and antibiotics were from HyClone (Logan, UT, USA). Transwell inserts with an 8 μm polycarbonate membrane and cell culture plastic labware were purchased from Corning Inc. (New York, NY, USA). Collagen IV was a product of Trevigen Inc. (Gaithersburg, MD, USA). Antibodies directed to Hsp90α (ab59459) and Hsp90β (ab53497) were purchased from Abcam (Cambridge, UK). Secondary Alexa 488-labeled conjugates were from Jackson ImmunoResearch Laboratories Inc. (West Grove, PA, USA). All other chemicals were from Sigma-Aldrich (St. Louis, MO, USA).

### 4.2. Cells and Virus

Human fibrosarcoma HT1080, human glioblastoma A-172, and BHK-21 cells were obtained from the Cell Culture Collection of Vertebrates (St. Petersburg, Russia). The cells were cultured in DMEM with 10% FBS (DMEM-FBS) and antibiotics. All cell lines were treated with a mycoplasma removal agent (MRA, Bio-Rad) to eliminate possible mycoplasma contamination. The laboratory strain Ka of the pseudorabies virus (PRV) was obtained from the collection of viruses at the Institute of Veterinary Medicine (Kiev, Ukraine). PRV was propagated on BHK-21 cells. The effect of 2,5-DHBA–gelatin on the adsorption of PRV to cells was determined by the virus plaque assay as described [31]. In brief, 24-well plates with monolayers of BHK-21 cell were chilled and washed with cold PBS. The 2,5-DHBA–gelatin polymer or heparin diluted in ice-cold DMEM and PRV (75–100 pfu) were simultaneously added to wells. The final concentrations of 2,5-DHBA–gelatin polymer or heparin were in the range of 1–50 µg/mL. Cells were incubated for 1 h at 4 °C, the conditions that prevent the penetration of the virus into cells, and washed with cold PBS to discard free PRV that did not attach to cells. Cells were covered with methylcellulose (0.5% in DMEM-FBS) and further incubated for 48 h at 37 °C. After that, BHK-21 cell monolayers were fixed with ethanol (−20 °C), and viral plaques were stained with the PRV-directed MAbs labeled with peroxidase and counted under an inverted microscope.

### 4.3. Preparation of the 2,5-DHBA–Gelatin Conjugate

The 2,5-DHBA–gelatin conjugate was synthesized by the laccase-catalyzed covalent coupling of 2,5-DHBA to gelatin [31]. The purification of laccase from the fungus *Cerrena unicolor* VKM F-3196 is described [56]. In brief, laccase (10 U/mL) was added to a solution containing 12.5 mg/mL of gelatin and 50 mM 2,5-DHBA in 50 mM Na-acetic buffer, pH 5.0, and the mixture was incubated for 15 h at 30 °C under stirring on a rotary shaker. The reaction was stopped by boiling, and the mixture was dialyzed against distilled water. The reaction mixture was centrifuged at 5000× *g* for 30 min, and the precipitate was discarded. The 2,5-DHBA–gelatin conjugate was concentrated using a centrifugal concentrator (Vivaspin 2, MWCO, 10 kDa).

### 4.4. Purification and Fluorescent Labeling of Hsp90

Native bovine Hsp90 was purified from bovine brains, as described earlier [57]. Bovine brains were obtained from a local slaughterhouse. The purity of Hsp90 amounted to 95%–98%. Purified Hsp90 consisted of both isoforms of Hsp90—Hsp90α and Hsp90β. For cell culture experiments, purified native Hsp90 was extensively dialyzed against DMEM. Purified native Hsp90 was labeled with FITC using the Hsp90/FITC molar ratio 1:4.

### 4.5. Determination of Cytotoxicity and Antiproliferative Activity of 2,5-DHBA–Gelatin

MTT assay was used for the evaluation of cytotoxic and antiproliferative effects of the 2,5-DHBA–gelatin polymeric conjugate toward HT1080 and A-172 cells [58]. For the evaluation of toxicity of the polymer, the cells were grown in 96-well plates until confluence. Then, the cells were incubated for 72 h at 37 °C in DMEM-FBS containing 2,5-DHBA–gelatin or sulfated glycosaminoglycans (heparin, dermatan sulfate, chondroitin sulfate A) at different concentrations. After that, MTT (5 mg/mL) was added to wells, and the cells were further incubated for 2 h at 37 °C. Formazan crystals were dissolved in DMSO, the optical density was measured using a plate reader iMax (Bio-Rad (Hercules, CA, USA)) (wavelength 550 nm), and the cytotoxicity of samples was calculated.

To evaluate the antiproliferative activity of the polymer, cells were diluted in DMEM-FBS containing 2,5-DHBA–gelatin or sulfated glycosaminoglycans at different concentrations and plated to the wells of 96-well plates (5.0–7.0 × 10^3^ cells per well). The cells were incubated for 48 h, stained with MTT as described above, and the inhibition of cell growth was determined. Geldanamycin was used as a positive control in the cytotoxicity and antiproliferative activity assays.

### 4.6. Evaluation of Basal Cell Migration and Invasion

The determination of basal cell migration and invasion was performed as described [30] with minor modifications. HT1080 and A-172 cells grown to 80%–90% confluence were starved by cultivation for 24 h in DMEM containing 0.2% BSA (DMEM-BSA). Then, cells were dissociated from plastic surface with 0.05% Na-EDTA and washed with DMEM-BSA. The cells were resuspended in DMEM-BSA in the absence and presence of DHBA–gelatin or sulfated glycosaminoglycans and seeded in the top chambers of inserts with a permeable membrane. For the assessment of invasion, the membranes were coated with collagen IV according to the manufacturer’s recommendations. DMEM supplemented with 5% FBS was added to bottom chambers to form a chemotactic gradient. The cells migrated through the polycarbonate membrane for 6 h in the migration assay and for 24 h in the invasion assay. The cells were removed from the upper side of the insert membrane with a cotton swab, and the cells attached to the bottom membrane were fixed with methyl alcohol and stained for 20 min with 1% crystal violet. Then, the fixed cells were lysed with acetic acid (10%), and the optical density was measured by the use of a plate reader at 595 nm (*OD*_595_). The spontaneous migration and invasion of cells without the chemotactic gradient were subtracted from *OD*_595_ values. It was assumed that the *OD*_595_ value of control untreated cells is 100%.

### 4.7. Hsp90-Stimulated Migration and Invasion of Cells

The Hsp90-stimulated migration and invasion assays were performed as described for basal migration/invasion with the exception that, together with the 2,5-DHBA–gelatin conjugate, native Hsp90 (50 μg/mL) was added to DMEM-BSA to stimulate cell migration/invasion. The *OD*_595_ values of control unstimulated cells without Hsp90 were subtracted from the *OD*_595_ values of cells stimulated with native Hsp90 in the presence of different polymers, and the residual was divided by the *OD*_595_ values of control cells. The Hsp90-mediated stimulation of migration and invasion of cells incubated with Hsp90 and without polymers was taken as 100%.

### 4.8. Determination of the Level of Membrane-Associated Hsp90α and Hsp90β

Cells in the exponential growth phase were incubated with the 2,5-DHBA–gelatin conjugate or with different sulfated glycosaminoglycans dissolved in DMEM-FBS for 1 h at 37 °C. Cells were dissociated from the plastic surface with 0.05% Na-EDTA before staining with primary Hsp90α- and Hsp90β-specific antibodies and secondary Alexa 488-labeled conjugates. All incubations were carried out at 4 °C in PBS containing 1% BSA and 0.05% NaN_3_ (PBS-BSA-NaN_3_) to exclude the internalization of antibodies. Stained cells were washed by gentle centrifugation and fixed for 15 min at 4 °C with 0.5% formaldehyde. The flow cytometry analysis was performed on an Accuri C6 flow cytometer (Becton Dickinson; Franklin Lakes, NJ, USA). Isotype controls were used in all experiments. A total of 50,000-200,000 events were collected for each analysis. The BD Accuri C6 software was used for the determination of the mean fluorescence intensity (MFI).

### 4.9. Binding of Hsp90 to Cells in the Presence of the 2,5-DHBA–Gelatin Conjugate

Cells were dissociated from the plastic surface with 0.05% Na-EDTA and washed with cold PBS-BSA-NaN_3_. Cells were incubated at 4 °C for 1 h with the Hsp90–FITC conjugate diluted in PBS-BSA-NaN_3_ containing 2,5-DHBA–gelatin or different sulfated glycosaminoglycans (50 μg/mL). After that, the cells were washed with cold PBS-BSA-NaN_3_, fixed with formaldehyde (0.5%, 15 min), and analyzed by flow cytometry.

### 4.10. Statistical Analysis

The results are expressed as the mean value ± SD. Statistical analyses were performed by the SigmaPlot 13.0 software (Systat Software Inc., San Jose, CA, USA) by use of the one-way ANOVA and the Dunnett’s post-hoc test. All experiments were carried out independently three to five times. Representative results are presented in the figures and tables.

## Figures and Tables

**Table 1:** Cytotoxic and antiproliferative activities of the 2,5-DHBA–gelatin conjugate.

Substance	Concentration, µg/mL	Cytotoxicity (%) ^(a)^		Antiproliferative Activity (%) ^(a)^	
		HT1080	A-172	HT1080	A-172
2,5-DHBA–gelatin	1000	104 ± 15	97 ± 12	102 ± 16	102 ± 13
	300	96 ± 12	96 ± 14	99 ± 14	101 ± 12
	100	103 ± 16	100 ± 13	104 ± 17	94 ± 17
	30	95 ± 13	93 ± 14	96 ± 17	96 ± 16
	10	97 ± 15	99 ± 16	93 ± 14	96 ± 15
Heparin	1000	104 ± 17	97 ± 14	95 ± 15	92 ± 15
	300	101 ± 13	99 ± 14	94 ± 12	98 ± 14
	100	101 ± 12	105 ± 15	99 ± 13	98 ± 14
	30	93 ± 14	96 ± 15	103 ± 15	102 ± 16
	10	98 ± 13	94 ± 13	101 ± 15	95 ± 11
Chondroitin sulfate	1000	100 ± 16	99 ± 12	91 ± 12	94 ± 16
	300	100 ± 12	103 ± 13	95 ± 14	92 ± 15
	100	104 ± 16	105 ± 13	93 ± 12	95 ± 15
	30	94 ± 14	101 ± 16	99 ± 12	100 ± 13
	10	99 ± 16	95 ± 16	100 ± 13	101 ± 13
Dermatan sulfate	1000	99 ± 17	102 ± 15	92 ± 13	91 ± 15
	300	103 ± 14	97 ± 13	96 ± 14	93 ± 14
	100	104 ± 18	100 ± 14	101 ± 13	95 ± 14
	30	101 ± 16	101 ± 15	95 ± 14	104 ± 14
	10	97 ± 14	96 ± 14	103 ± 15	107 ± 17
Geldanamycin ^(b)^	5.6	5 ± 3	0	0	0
	0.56	43 ± 9	3 ± 3	13 ± 7	0
	0.056	94 ± 11	13 ± 4	55 ± 12	2 ± 2
	0.0056	98 ± 15	78 ± 14	100 ± 16	57 ± 8

^(a)^ The mean OD_550_ values for cells treated with different substances relative to control untreated cells, expressed as percentage, are presented. The mean OD_550_ value of control untreated cells was taken as 100%. The mean values of four to six repeats ± SD are presented. ^(b)^ Geldanamycin, which exhibits cytotoxic and antiproliferative activities, was used as a positive control.
